# Using Animal Models for Gender-Affirming Hormone Therapy

**DOI:** 10.1210/jendso/bvad144

**Published:** 2023-11-20

**Authors:** Krisha Aghi, Teddy G Goetz, Daniel R Pfau, Simón(e) D Sun, Eartha Mae Guthman, Troy A Roepke

**Affiliations:** Department of Integrative Biology and Physiology, University of California, Los Angeles, Los Angeles, CA 90095, USA; Department of Psychiatry, University of Pennsylvania, Philadelphia, PA 19104, USA; Department of Obstetrics and Gynecology, University of Michigan, Ann Arbor, MI 48109, USA; Department of Biomedical Engineering, University of Michigan, Ann Arbor, MI 48109, USA; Division of Pediatric Endocrinology, Diabetes and Metabolism, University of Michigan, Ann Arbor, MI 48109, USA; Cold Spring Harbor Laboratory, Cold Spring Harbor, NY 11724, USA; Center for Applied Transgender Studies, Chicago, IL, USA; Center for Applied Transgender Studies, Chicago, IL, USA; Princeton Neuroscience Institute, Princeton University, Princeton, NJ 08540, USA; Department of Animal Sciences, School of Biological and Environmental Sciences, Rutgers University, New Brunswick, NJ 08901, USA

**Keywords:** gender identity, gender-affirming hormone therapy, steroids, estrogens, testosterone, animal models

## Abstract

We have recently proposed experimental design guidelines and areas of study for preclinical rodent models of gender-affirming hormone therapy in neuroscience. These guidelines also apply to any field subject to the influences of gonadal steroid hormones, including metabolism and growth, cancer, and physiology. This perspective briefly describes our suggestions for these fields. Studying the effects of exogenous steroid hormones will have translational benefits for the community. We also discuss the need for equitable practices for cisgender scientists who wish to implement these guidelines and engage with the community. It is necessary that community-informed practices are implemented in preclinical research to maximize the benefit to transgender, nonbinary, and/or gender diverse (TNG) healthcare, which is currently in jeopardy in the United States, Europe, and across the globe.

The gonadal steroid hormones estrogen, progesterone, and testosterone have been used by people for sex/gender transition since their initial discovery approximately a century ago. In the decades since, the use of these hormones as gender-affirming hormone therapy (GAHT) became widespread among transgender individuals for biomedical sex/gender transition (for historical reviews of hormonal and other treatments of transgender children and adults, see [1]. Although the precise influences of gonadal steroid hormones on mental health are unclear (and likely dependent on social and environmental context), GAHT is clearly associated with improvements in mental health—regardless of formulation [2]. Nevertheless, there remains a dearth of research into the mechanisms by which GAHT improves mental health outcomes. This lack of research is used by anti-transgender activists to justify discriminatory practices and limit access to or outright deny care. An increasing number of states in the USA have enacted new laws and regulations limiting the ability of transgender children to access GAHT, which *de facto* forces some children to medically detransition [3]. As of June 2023, the number of states limiting access to care has increased to 20. In some states, implementation of laws banning GAHT for children also result in *de facto* bans for adult GAHT [4]. The current sociocultural and geopolitical moment, in combination with the difficulty of mechanistic neuroendocrine studies in humans, points to an urgent need for well-designed preclinical studies.

To address this exigent issue, we recently published a review outlining how to best design such studies [5]. Studies on the influence of estrogen-based GAHT (E-GAHT)/testosterone-based GAHT (T-GAHT) on physiological or neurological processes should be evaluated on whether “classic” endocrinological techniques (gonadectomy with surgical pellet implantation) or new GAHT models are most appropriate. In our review, we proposed best practices for experimental design that recapitulate the human experience of GAHT, such as comparing intact rodents with gonadectomized rodents, applying steroid replacement to both, and treating with androgen receptor blockers commonly used in E-GAHT. This work should focus on the needs of transgender populations. Here, we provide a brief overview of our recommendations for preclinical rodent models of GAHT and emphasize how this research should be conducted to address the material health needs of transgender populations.

## Receptors and Gene Expression

Gonadal hormones (estrogens, progestins, and androgens) exert their effects by acting as ligands to their cognate receptors. The most well-studied of the hormone receptors are the canonical nuclear receptors: estrogen receptor alpha and beta (ERα, ERβ), androgen receptor (AR), and progesterone receptors (PR-A, PR-B). These receptors are expressed at varying levels throughout the brain, with notably denser expression in subcortical regions important for social behavior, stress responses, metabolism, mood/affect, memory, and cognition. Therefore, understanding the mechanisms by which gonadal hormones influence these aspects of neural function is fundamental for improving GAHT.

Nuclear hormone receptors regulate gene expression to drive changes in neural circuit wiring, synaptic strength, and neural activity. When bound with their ligand, these receptors interact with the genome, acting as transcription factors and modifying chromatin profiles [6]. Recent research indicates that sex variability in neuronal gene expression is reliant on the acute hormonal environment, suggesting a potential mechanism by which GAHT can flexibly influence neuronal function throughout an individual's lifetime [7]. Additionally, gonadal hormones can act on nonneuronal populations, including microglia and astrocytes and may contribute to the mental health benefits associated with GAHT. Future research that links hormone-dependent neuronal activity changes with hormone-dependent gene regulation could reveal molecular pathways for personalized GAHT.

Membrane-bound hormone receptors are found on both the plasma membrane and the surface of organelles. Once ligand-bound, these receptors are often thought to initiate molecular cascades, leading to changes in cell function and behavior. The timescale of action is hypothesized to lead to parallel mechanisms of intracellular action in concert with nuclear receptor activation. Future research on GAHT could illuminate the interplay between membrane-bound receptors and nuclear receptors and interrogate how each receptor-type distinctly affects cellular function in multiple tissue-types.

## Cognition/Memory

Cognition, memory, and learning are influenced by gonadal hormones, and many of the brain regions involved in cognition express steroid receptors. However, evidence that estrogen replacement therapies can protect against cognitive decline remains controversial. Studies in cisgender humans partially support the hypothesis that steroids influence cognition and memory in older adults across sex. The influence of GAHT on cognitive measures in transgender, nonbinary, and/or gender diverse (TNG) people—in youth seeking to delay puberty or in older TNG individuals with years of GAHT—remains largely unknown. More clinical and preclinical studies are needed to assess the mechanisms of GAHT on cognition and memory and the interactions of GAHT and social/minority stressors in young and aging TNG populations.

## Mood/Stress Disorders

Hormonal milieus interacting with multiple brain regions lead to differences in the etiology, symptomology, and effectiveness of treatments for mood and stress disorders. Evidence that GAHT alters stress reactivity in clinical settings suggests that it has direct actions on stress circuitry, which may impact the mental health needs of individuals treated with GAHT. However, studies examining GAHT-associated mental health run the risk of pathologizing GAHTs. Therefore, we propose 2 lines of inquiry to pursue through animal models of mood and stress disorders. The first compares GAHT-treated animals to untreated controls, which may identify the direct action of GAHT on mood and stress-related brain regions. The second examines the mental health outcomes using animal models of mood and stress disorder by comparing GAHT-treated animals differentially exposed to an additional variable. Gender-based discrimination and perception of the contemporary geopolitical climate are related to elevated biomarkers of allostatic load in transgender populations. Such factors can be conceived as chronic psychosocial stressors; as such, particular focus should be given to rodent models of chronic psychosocial stress.

## Metabolism

Although steroidal hormones and their effects on general metabolism and physiology are historically well-studied, much remains to be studied about GAHT and its long-term outcomes on growth, overall metabolism, and bone health. Both estrogens and androgens are thought to regulate feeding behaviors, homeostatic control of thermoregulation, and overall body mass. However, as few studies have examined the effects of E-GAHT and T-GAHT on long-term body composition and growth, we propose preclinical studies to understand how varying regimens of GAHT lead to changes in body weight and the localization of fat accumulation before and after GAHT. These studies would allow for further tailoring of GAHT regimens to increase satisfactory transition outcomes in TNG populations. Additionally, bone health is thought to be regulated in part by circulating steroidal hormones. Age-dependent bone loss is partially attributed to a dysregulation of gonadal steroids. To address this, we propose longitudinal studies that examine the effects of GAHT on bone health in older TNG individuals and the maintenance of bone growth in young and middle-aged TNG individuals.

## Cancer

While much of our focus has been on neurobiology, our models can be easily applied to other endocrine-sensitive physiological and pathophysiological processes, such as cancer. While much has been written about health disparities in the TNG community in the context of cancer, the influence of GAHT on the prevalence of or risk for endocrine cancers is understudied. Furthermore, the mechanisms underlying these effects can only be postulated due to the lack of preclinical studies in animal and cellular models. Questions that can be addressed by the proposed models include both short-term and long-term treatment with both E-GAHT and T-GAHT in young adult and aged rodents, activation of cellular processes by both treatment types on ER/PR-positive breast cancer cells, the influence of E-GAHT on prostate cancers, and the long-term effects of pubertal delay on endocrine cancers, including intestinal and reproductive cancers.

## Conclusions

We must recognize that animal GAHT models are limited in their ability to fully study the uniquely human experience of gender-affirming treatments. However, these models can improve our knowledge of how GAHTs influence physiological and neurological processes. It is imperative to understand that GAHT and the social stressors discussed could have reciprocal influences on their actions and should be individually and synergistically studied. There remain significant barriers to healthcare and to STEM careers for TNG people, which are amplified by current political decisions. As such, studies using the proposed preclinical models of GAHT must incorporate collaborations with TNG community members, either in the lab or through community outreach. As transgender, nonbinary, and gender diverse identities are increasingly common, the prevalence of binary classifications significantly hinders meaningful research, particularly when applied to the human condition. The TNG community has identified GAHT research priorities for GAHT [8] to ensure study outcomes and interpretations that align with TNG needs and experiences. Our proposed preclinical GAHT mouse models can create beneficial relationships between science/healthcare and the TNG community, but only when appropriately applied and with direct involvement of the TNG community.

## Disclosures

T.A.R. is an Editorial Board member for the *Journal of the Endocrine Society*. The remaining authors have nothing to disclose.

## Data Availability

There are no data in this manuscript to share.

## Abbreviations

E-GAHT: estrogen-based gender-affirming hormone therapy
ER: estrogen receptor
GAHT: gender-affirming hormone therapy
PR: progesterone receptor
T-GAHT: testosterone-based gender-affirming hormone therapy
TNG: transgender, nonbinary, and/or gender diverse
